# Structural Analysis of the SARS-CoV-2 Omicron Variant Proteins

**DOI:** 10.34133/2021/9769586

**Published:** 2021-12-28

**Authors:** Qiangzhen Yang, Ali Alamdar Shah Syed, Aamir Fahira, Yongyong Shi

**Affiliations:** ^1^Bio-X Institutes, Key Laboratory for the Genetics of Developmental and Neuropsychiatric Disorders (Ministry of Education), Shanghai Jiao Tong University, 1954 Huashan Road, Shanghai 200030, China; ^2^Biomedical Sciences Institute of Qingdao University (Qingdao Branch of SJTU Bio-X Institutes), Qingdao University, Qingdao 266003, China; ^3^Shanghai Key Laboratory of Psychotic Disorders, Shanghai Mental Health Center, Shanghai Jiao Tong University School of Medicine, Shanghai 200030, China; ^4^Shanghai Key Laboratory of Sleep Disordered Breathing, Shanghai Jiao Tong University Affiliated Sixth People's Hospital, Shanghai, China; ^5^The First Affiliated Hospital of Zhengzhou University, Zhengzhou 450052, China; ^6^Department of Psychiatry, First Teaching Hospital of Xinjiang Medical University, Urumqi, 830046, China

## Abstract

The spread of the latest SARS-CoV-2 variant Omicron is particularly concerning because of the large number of mutations present in its genome and lack of knowledge about how these mutations would affect the current SARS-CoV-2 vaccines and treatments. Here, by performing phylogenetic analysis using the Omicron spike (S) protein sequence, we found that the Omicron S protein presented the longest evolutionary distance in relation to the other SARS-CoV-2 variants. We predicted the structures of S, M, and N proteins of the Omicron variant using AlphaFold2 and investigated how the mutations have affected the S protein and its parts, S1 NTD and RBD, in detail. We found many amino acids on RBD were mutated, which may influence the interactions between the RBD and ACE2, while also showing the S309 antibody could still be capable of neutralizing Omicron RBD. The Omicron S1 NTD structures display significant differences from the original strain, which could lead to reduced recognition by antibodies resulting in potential immune escape and decreased effectiveness of the existing vaccines. However, this study of the Omicron variant was mainly limited to structural predictions, and these findings should be explored and verified by subsequent experiments. This study provided basic data of the Omicron protein structures that lay the groundwork for future studies related to the SARS-CoV-2 Omicron variant.

## 1. Main

The prolonged and extensive spread of SARS-CoV-2 has induced some unexpected mutations that can boost virus transmission and disease severity [1]. A new SARS-CoV-2 variant of B.1.1.529 was identified in South Africa on 24 November 2021, and the World Health Organization (WHO) subsequently designated it, B.1.1.529, as a variant of concern (VOC) and named it Omicron (http://www.who.int/). The latest SARS-CoV-2 variant is particularly concerning because of the large number of mutations present in its genome, and how so little is known about the variant.

**Figure 1:**
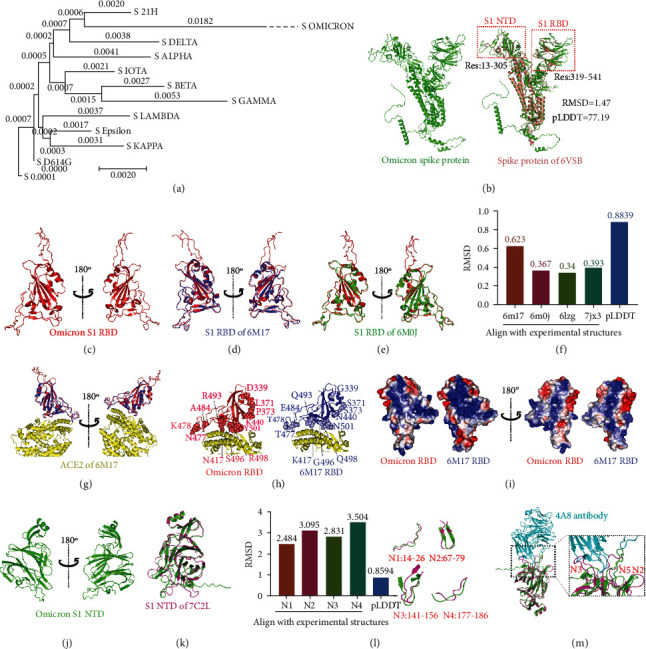
(a) The phylogenetic analysis of all SARS-CoV-2 variants based on the spike protein sequence. The sequences were downloaded from GSAID (http://nextstrain.org/sars-cov-2). (b) The predicted structure of the Omicron variant. The comparison of spike protein between the Omicron variant and experimental structure (PDB: 6VSB). (c) The S1 RBD structure of Omicron variant. (d) The alignment of S1 RBD structure between Omicron variant and experimental structure (PDB: 6M17). (e) The alignment of S1 RBD structure between Omicron variant and experimental structure (PDB: 6M0J). (f) The RMSD values of S1 RBD between Omicron variant and experimental structures (PDB: 6M17, 6M0J, 6LZG, and 7JX3). The pLDDT value of the predicted S1 RBD structure was divided by 100. (g) The analysis of interactions between S1 RBD and ACE2. Red and blue indicate the S1 RBD of Omicron and 6M17, respectively. (h) The amino acid mutations on the Omicron S1 RBD. (i) The comparison of surface charge properties on S1 RBD between the Omicron variant and the experimental structure (PDB: 6M17). Red and blue colors on the surface indicate negative and positive charges, respectively. (j) The S1 NTD structure of the Omicron variant. (k) The comparison of S1 NTD between the Omicron variant and the experimental structure (PDB: 7C2L). (l) The RMSD values of the loop N1-4 between NTD and experimental structure (PDB: 7C2L). The pLDDT value of the predicted S1 NTD of the Omicron variant was divided by 100. The structural comparison of the S1 NTD loop N1-4 can be seen displayed on right. (m) The analysis of interactions between S1 NTD with 4A8 antibody (PDB: 7C2L).

To detect the evolutionary relationship between Omicron and the rest of the SARS-CoV-2 variants, we performed the phylogenetic analysis of all SARS-CoV-2 variants based on the spike (S) protein sequence. The Omicron S protein presented the longest evolutionary distance than other variants (Figure 1(a)). The multiple sequence alignment of present VOC variants showed that the Omicron S protein contains thirty-four amino acid (AA) mutations, including A67V, H69-, V70-, T95I, G142-, V143-, Y144-, Y145D, N211-, L212I, G339D, S371L, S373P, K417N, N440K, G446S, S477N, T478K, E484A, Q493R, G496S, Q498R, N501Y, T547K, D614G, H655Y, N679K, P681H, N764K, D796Y, N856K, Q954H, N969K, and L981F (Figure S1). These results clearly show that the Omicron S protein is significantly altered compared to previous SARS-CoV-2 variants.

The SARS-CoV-2 S protein is important for mediating entry into host cells and is the main target of neutralizing antibodies [2]; the structure of the S protein is an essential characteristic of any variant and obtaining this structure helps us better understand the Omicron variant. AlphaFold2 is an open-source computational approach developed to help us acquire accurate protein structures based on genetic data [3]. Here, we employed AlphaFold2 to predict the Omicron variants S protein structure, which displayed a low root means square deviation (RMSD) value of 1.47 compared with the experimental structure (PDB: 6VSB [4]) and a high predicted local distance difference test (pLDDT) value of 77.19 (Figure 1(b)). Given that the pLDDT value calculated by AlphaFold2 is above 70, the prediction can be considered highly confident and accurate [3]. The theoretical isoelectric point (pI)/molecular weight (Mw) of Omicron S protein is 7.34/140973.70. The comparison of S protein structure between Omicron and experimental structure of 6VSB showed the main difference lies on the N-terminal domain (NTD) and receptor-binding domain (RBD) of S protein subunit 1 (S1) (Figure 1(b) red boxes). Thus, we further investigated both the S1 RBD and NTD (Figures 1(c) and 1(j)) structures, respectively.

The resulting pLDDT value of 88.93 indicates that the predicted Omicron S1 RBD was also highly accurate (Figure 1(f)). The Omicron S1 RBD structure showed high similarities with the experimental structures (PDB: 6M17 and 6M0J) (Figures 1(d) and 1(e)), and the comparison of Omicron S1 RBD with experimental structures (PDB: 6M17[5], 6M0J [6], 6LZG [7], and 7JX3 [8]) showed low RMSD values (Figure 1(f)), which reflected that the amino acid (AA) mutations on the Omicron S1 RBD could slightly influence its structure. Given that the S1 RBD could bind to angiotensin-converting enzyme 2 (ACE2) [9], we analyzed the interactions between Omicron S1 RBD and ACE2. The Omicron receptor-binding motif (RBM) structure is highly similar to the experimental structures of original strain (Figures 1(c)–1(f)), which indicated that the interactions between RBM might be slightly influenced. The distances between interaction points of Omicron RBD and ACE2 were not significantly changed compared to the RBD-ACE2 complex of the original strain (PDB: 6M17) (Figure 1(g)). The results suggested that interactions between Omicron RBD and ACE2 might be slightly altered compared with the original strains. Moreover, some amino acids on RBD interacting with ACE2 were mutated, such as K417N, N440K, Q493R, G496S, Q498R, and N501Y (Figure 1(h)). The AA mutations of K417N, N440K, Q493R, and Q498R could change the charge characteristics of amino acids (Figure 1(i)), which may influence the interactions between the RBD and ACE2. Notably, we found the conserved RBD epitope recognized by antibody S309 was not changed in the Omicron RBD (Figure S2 A); therefore, S309 could still potentially be used in the treatment of the SARS-CoV-2 Omicron variant [10].

However, the Omicron S1 NTD structure showed that many epitopes that could be recognized by various antibodies are altered in comparison to the original strain (PDB: 7C2L [11]) (Figure 1(k)). This result displayed a high pLDDT value of 85.94 indicated that the predicted Omicron S1 NTD structure was accurate (Figure 1(l)). The four loops on NTD that are important for recognition of antibody showed the significant difference between Omicron and structure of 7C2L, as shown by their high RMSD values (Figure 1(l)). Furthermore, we analyzed the interactions between antibody 4A8 and Omicron S1 NTD and found that the epitope targeted by 4A8 was significantly changed, which suggested that the effectiveness of 4A8 and other antibodies targeting this epitope could be reduced. The mutations on the Omicron S1 NTD may increase immune escape rates and decrease the effectiveness of the existing vaccines. In other words, these mutations on S1 NTD are worthy of attention.

Moreover, we also predicted the structures of the membrane (M) protein and nucleocapsid (N) using Alphafold2. We acquired the highly accurate membrane protein structure with a pLDDT value of 82.84 and the low accurate nucleocapsid structure with a pLDDT value of 69.1 (Figure S2 B and C). The nucleocapsid structure may be insufficient for further investigation, but the predicted membrane protein structure is accurate enough to be suitable for subsequent functional analyses. The inability of AlphaFold2 to accurately predict the structure of the nucleocapsid is likely due to its high structural complexity that is resulted from its diversified interactions with the viral RNA. The theoretical pI/Mw of Omicron N and M proteins are 10.09/45340.48 and 9.51/25119.59, respectively.

In conclusion, the Omicron S protein presented the longest evolutionary distance than other variants. We presented the structures of S, M, and N proteins of the newly identified SARS-CoV-2 Omicron variant and investigated the changes of S protein and its parts, S1 NTD and RBD, in detail. We found many amino acids on RBD were mutated, which may influence the interactions between the RBD and ACE2, while the S309 antibody could still be effective in neutralizing Omicron RBD. The Omicron S1 NTD structures display significant differences from the original strain, which could lead to reduced recognition by antibodies resulting in potential immune escape and decreased effectiveness of the existing vaccines. Taken together, the mutations on S protein might increase the immune escape and transmissibility of the Omicron variant. However, this study of the Omicron variant was mainly limited to structural predictions, and these findings should be explored and verified by subsequent experiments. The present study provided basic data of the Omicron protein structures that lay the groundwork for future studies related to the SARS-CoV-2 Omicron variant.

## Supplementary Materials

Supplementary Materials Figure S1: the multiple sequence alignment analysis on the spike protein of VOC variants. Figure S2: (A) the interactions between S309 antibody with Omicron RBD. (B-C) The structures of M (B) and N (C) proteins. The protein structural files predicted by AlphaFold were submitted as supplemental materials.
