# Supplementary material for: Structural Analysis of the SARS-CoV-2 Omicron Variant Proteins
Source: Research (Wash D C). 2021 Dec 28;2021:9769586. doi: 10.34133/2021/9769586 (PMC8765807; doi:10.34133/2021/9769586)
Supplement: Supplementary Materials — Figure S1: the multiple sequence alignment analysis on the spike protein of VOC variants. Figure S2: (A) the interactions between S309 antibody with Omicron RBD. (B-C) The structures of M (B) and N (C) proteins. The protein structural files predicted by AlphaFold were submitted as supplemental materials. [file 9769586.f1.docx]

**Methods**

**1. Structure modeling with AlphaFold**

The structural prediction of spike protein was based on the model of AlphaFold v2.0 entered in CASP14 and published in Nature [1]. The inference pipeline and source code of AlphaFold are available under an open-source license at https://github.com/deepmind/alphafold. The parameter of preset is the same as that used in CASP14, which runs with all genetic databases and with 8 ensembles. The mirrored databases used in our study include BFD [2], MGnify clusters [3], UniRef90 [4], Uniclust30 [5], protein data bank (PDB), and PDB70 [6]. The max date of the databases was set to 2021-11-25. The spike protein sequences of different SARS-CoV-2 variants downloaded from GISAID (http://gisaid.org) were used as inputs. The graphics processing unit (GPU) used in this study was NVIDIA Tesla V100 on the π 2.0 cluster supported by the Center for High-Performance Computing at Shanghai Jiao Tong University.

**2. Comparisons between experimental and predicted structures**

All the experimental protein structures used in this study were downloaded from PDB. The 3D protein structures were visualized by the software PyMol. The similarity between experimental and predicted structures was evaluated by the root-mean-square deviation (RMSD) and the predicted local-distance difference test (pLDDT). The RMSD and pLDDT between experimental and predicted structures were calculated by the software PyMol and AlphaFold, respectively [7, 8].

**3. Cluster analyses of spike proteins of different SARS-CoV-2 variants**

The cluster analyses were performed based on both protein sequences and structures. The software Molecular Evolutionary Genetics Analysis-X (MEGA-X) was used for phylogenetic analyses based on the spike protein sequences. The ClustalW alignment algorithms were used to align the multiple sequences, and the evolutionary tree was generated using the maximum likelihood method [9]. The multiple sequence alignment analysis was performed based on the online servers of Clustal Omega [10] and ESPript V3.0 [11]. The pI/Mw of Omicron S, N, and M proteins using the online server of ExPASy [12-14] (web.expasy.org/cgi-bin/compute_pi/pi_tool).


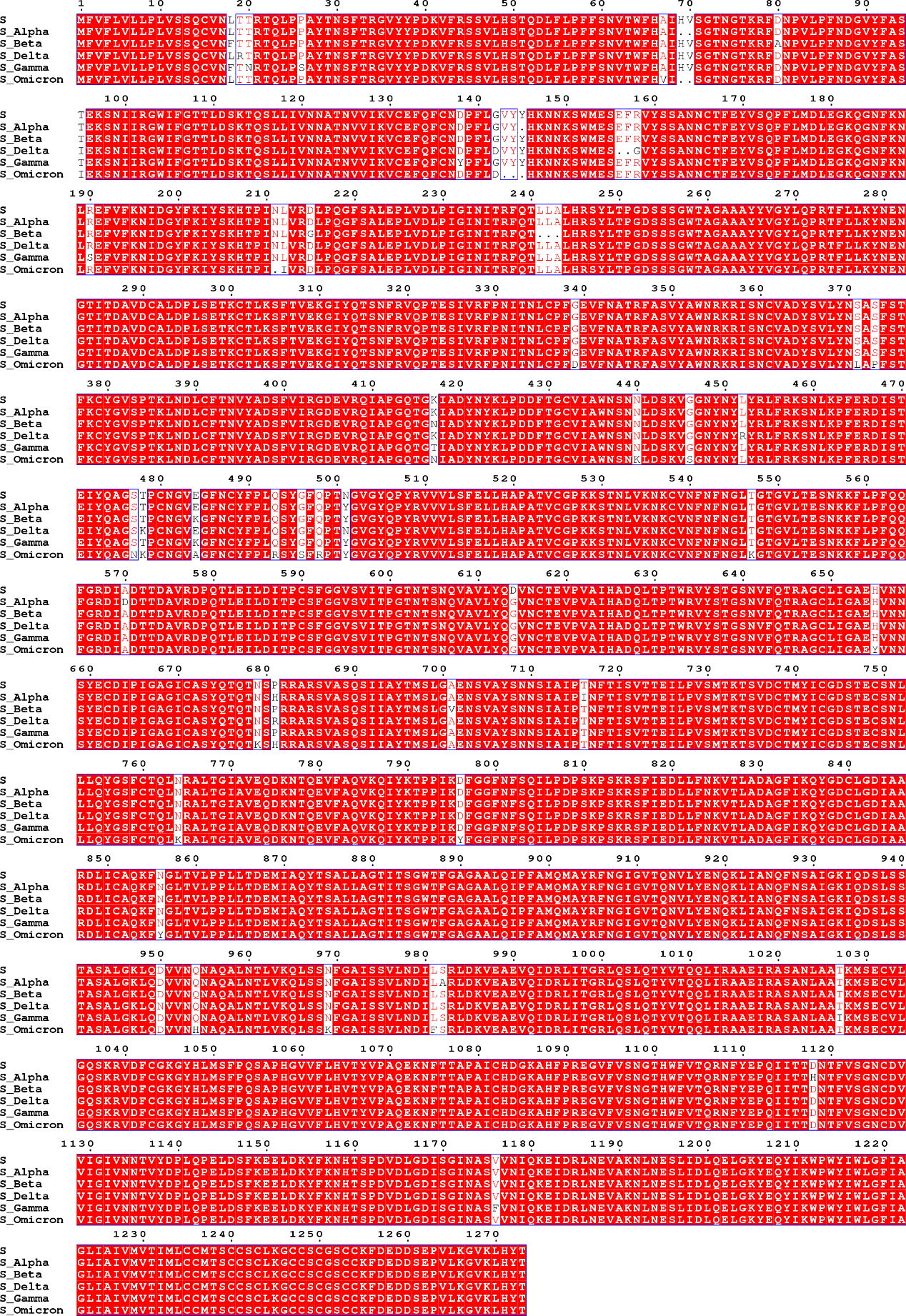


Figure S1. The multiple sequence alignment analysis on the spike protein of VOC variants. The spike protein sequence of the Omicron variant was downloaded from nextstrain.org/ncov/gisaid/global (ID: NICD-N21605-DX64490).


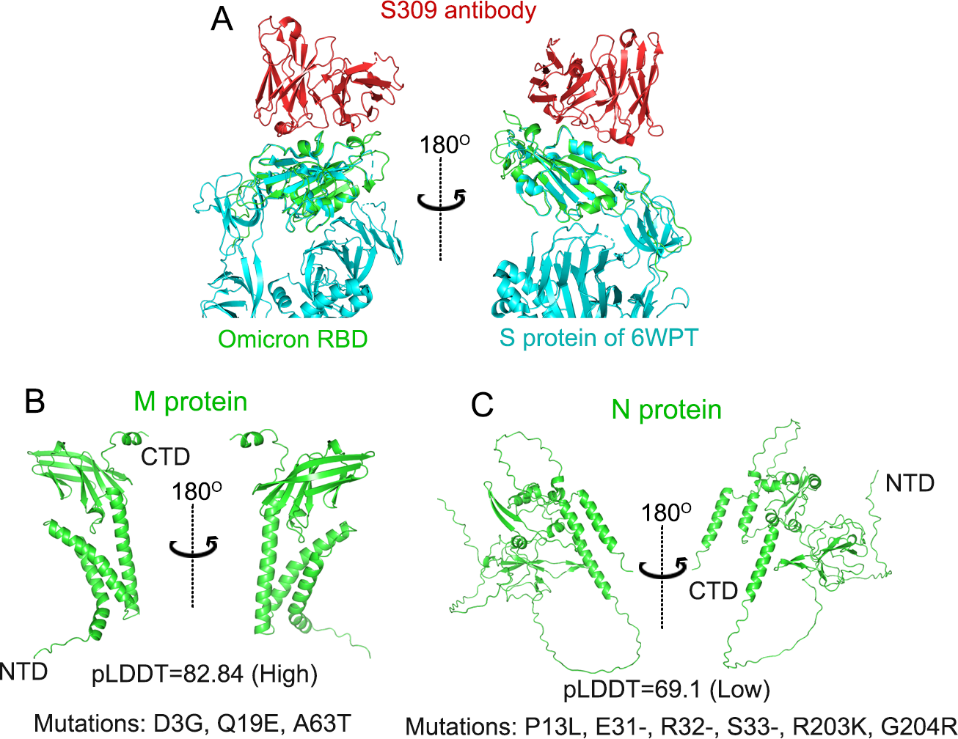


Figure S2. (A) The interactions between S309 antibody with Omicron RBD. (B-C) The structures of M (B) and N (C) proteins.

**Data availability**

All the data is available on RCSB and UniProt, and any simulation data will be provided on demand. The protein structural files predicted by AlphaFold were submitted as supplemental materials. The spike protein sequence of the Omicron variant was downloaded from nextstrain.org/ncov/gisaid/global (ID: NICD-N21605-DX64490).

**References:**

1. Jumper, J., et al., *Highly accurate protein structure prediction with AlphaFold.* Nature, 2021. **596**(7873): p. 583-589.

2. Steinegger, M., M. Mirdita, and J. Söding, *Protein-level assembly increases protein sequence recovery from metagenomic samples manyfold.* Nature Methods, 2019. **16**(7): p. 603-606.

3. Mitchell, A.L., et al., *MGnify: the microbiome analysis resource in 2020.* Nucleic Acids Research, 2019. **48**(D1): p. D570-D578.

4. Steinegger, M. and J. Söding, *Clustering huge protein sequence sets in linear time.* Nature Communications, 2018. **9**(1): p. 2542.

5. Mirdita, M., et al., *Uniclust databases of clustered and deeply annotated protein sequences and alignments.* Nucleic Acids Research, 2016. **45**(D1): p. D170-D176.

6. Berman, H.M., et al., *The Protein Data Bank.* Nucleic Acids Research, 2000. **28**(1): p. 235-242.

7. Tunyasuvunakool, K., et al., *Highly accurate protein structure prediction for the human proteome.* Nature, 2021. **596**(7873): p. 590-596.

8. Mariani, V., et al., *lDDT: a local superposition-free score for comparing protein structures and models using distance difference tests.* Bioinformatics, 2013. **29**(21): p. 2722-2728.

9. Madeira, F., et al., *The EMBL-EBI search and sequence analysis tools APIs in 2019.* Nucleic Acids Res, 2019. **47**(W1): p. W636-w641.

10. Madeira, F., et al., *The EMBL-EBI search and sequence analysis tools APIs in 2019.* Nucleic acids research, 2019. **47**(W1): p. W636-W641.

11. Robert, X. and P. Gouet, *Deciphering key features in protein structures with the new ENDscript server.* Nucleic Acids Research, 2014. **42**(W1): p. W320-W324.

12. Bjellqvist, B., et al., *The focusing positions of polypeptides in immobilized pH gradients can be predicted from their amino acid sequences.* Electrophoresis, 1993. **14**(10): p. 1023-31.

13. Bjellqvist, B., et al., *Reference points for comparisons of two-dimensional maps of proteins from different human cell types defined in a pH scale where isoelectric points correlate with polypeptide compositions.* Electrophoresis, 1994. **15**(3-4): p. 529-39.

14. Wilkins, M.R., et al., *Protein Identification and Analysis Tools in the ExPASy Server*, in *2-D Proteome Analysis Protocols*, A.J. Link, Editor. 1999, Humana Press: Totowa, NJ. p. 531-552.
